# Development of Gel-Filter Method for High Enrichment of Low-Molecular Weight Proteins from Serum

**DOI:** 10.1371/journal.pone.0115862

**Published:** 2015-02-27

**Authors:** Lingsheng Chen, Linhui Zhai, Yanchang Li, Ning Li, Chengpu Zhang, Lingyan Ping, Lei Chang, Junzhu Wu, Xiangping Li, Deshun Shi, Ping Xu

**Affiliations:** 1 State Key Laboratory of Proteomics, National Engineering Research Center for Protein Drugs, Beijing Proteome Research Center, National Center for Protein Sciences, Beijing Institute of Radiation Medicine, Beijing, 102206, P. R. China; 2 State Key Laboratory for Conservation and Utilization of Subtropical Agro-Bioresources, Guangxi University, Nanning, 530005, P. R. China; 3 Department of Biochemistry, School of Medicine, Wuhan University, Wuhan, 430071, P. R. China; 4 Key Laboratory of Combinatorial Biosynthesis and Drug Discovery (Wuhan University), Ministry of Education, and Wuhan University School of Pharmaceutical Sciences, Wuhan, 430071, P. R. China; Shantou University Medical College, CHINA

## Abstract

The human serum proteome has been extensively screened for biomarkers. However, the large dynamic range of protein concentrations in serum and the presence of highly abundant and large molecular weight proteins, make identification and detection changes in the amount of low-molecular weight proteins (LMW, molecular weight ≤ 30kDa) difficult. Here, we developed a gel-filter method including four layers of different concentration of tricine SDS-PAGE-based gels to block high-molecular weight proteins and enrich LMW proteins. By utilizing this method, we identified 1,576 proteins (n = 2) from 10 μL serum. Among them, 559 (n = 2) proteins belonged to LMW proteins. Furthermore, this gel-filter method could identify 67.4% and 39.8% more LMW proteins than that in representative methods of glycine SDS-PAGE and optimized-DS, respectively. By utilizing SILAC-AQUA approach with labeled recombinant protein as internal standard, the recovery rate for GST spiked in serum during the treatment of gel-filter, optimized-DS, and ProteoMiner was 33.1 ± 0.01%, 18.7 ± 0.01% and 9.6 ± 0.03%, respectively. These results demonstrate that the gel-filter method offers a rapid, highly reproducible and efficient approach for screening biomarkers from serum through proteomic analyses.

## Introduction

Serum contains a highly complex mixture of proteins/peptides secreted from tissues and organs throughout the body, which systemically reflects the physiological or pathological states of a living organism [1]. Identification and quantitation of these proteins/peptides can provide valuable information for diagnosis and prognosis in health care [2,3].

In the past decade, mass spectrometry (MS) has been developed as a proteomics platform for the sequencing and screening of potential disease-related biomarkers by comparing serum samples from healthy individuals and patients [4]. However, proteomic characterization using MS is challenging, because the dynamic range of proteins/peptides in serum is about 2–3 orders of magnitude broader (10^10∼11^) than that in regular samples, and higher abundant peptides/proteins are preferentially sequenced by MS [5,6]. In addition, there are 22 abundant and well-characterized proteins in serum, including albumin, immunoglobulin, fibrinogen, alpha 1-antitrypsin, alpha 2-macroglobulin, which together represent 99% of total serum protein [7,8]. Some proteins, such as albumin, can even reach concentrations of 35–50 mg/mL in serum [9]. The remaining 1% of the other protein component in serum is composed of thousands of low-abundance and low-molecular weight (LMW, MW ≤ 30 kDa) proteins/peptides, including peptide hormones, cytokines, growth factors, as well as proteins/peptides originating from normal cell or tissue leakage as a result of cell death or damages and proteolytic fragments of larger proteins [9]. From a diagnostic point of view, these proteins/peptides are even more important than the abundant ones to reflect valuable disease-related information [10]. For example, the low-abundance cellular and tissue hormone leptin, with the molecular weight of 16 kDa, is supposedly involved in the regulation of food intake and metabolism [11]. In recent years, many researchers have shown that leptin is closely involved in the occurrence and development of diseases, such as obesity [12,13] and diabetes mellitus [14,15].

In order to analyze these low-abundance and LMW proteins in complicated serum samples, various methods have been developed in the past decade, such as ultrafiltration [8,16], organic solvent precipitation [17,18], electrophoresis [19], chromatography (on-column or on magnetic beads) [20–22] and affinity depletion[23]. The reduction of sample complexity is essentially the first step. However, these pre-fractionation strategies have limited efficiency in the depletion of highly abundant proteins (HAPs), resulting in potential loss of components through binding to the high-molecular weight (HMW) proteins and consequent unsatisfactory detection of LMW proteins.

ProteoMiner, also called combinatorial solid-phase library, is one of the widely used and efficient strategies for low abundant proteins by decreasing the high abundant proteins in complicated biological samples, such as serum[24], urine[25], platelets[26]. This combinatorial solid-phase library contains large population of beads binding with peptides with high diversity, in which each bead contains millions of unique hexapeptide ligands. The HAPs have saturation limitation of binding sites, whereas those remaining unbound or weakly bound proteins were washed away in the flow-through. In contrast, bindable low-abundance proteins can be enriched on their corresponding beads[27].

**Fig 1:**
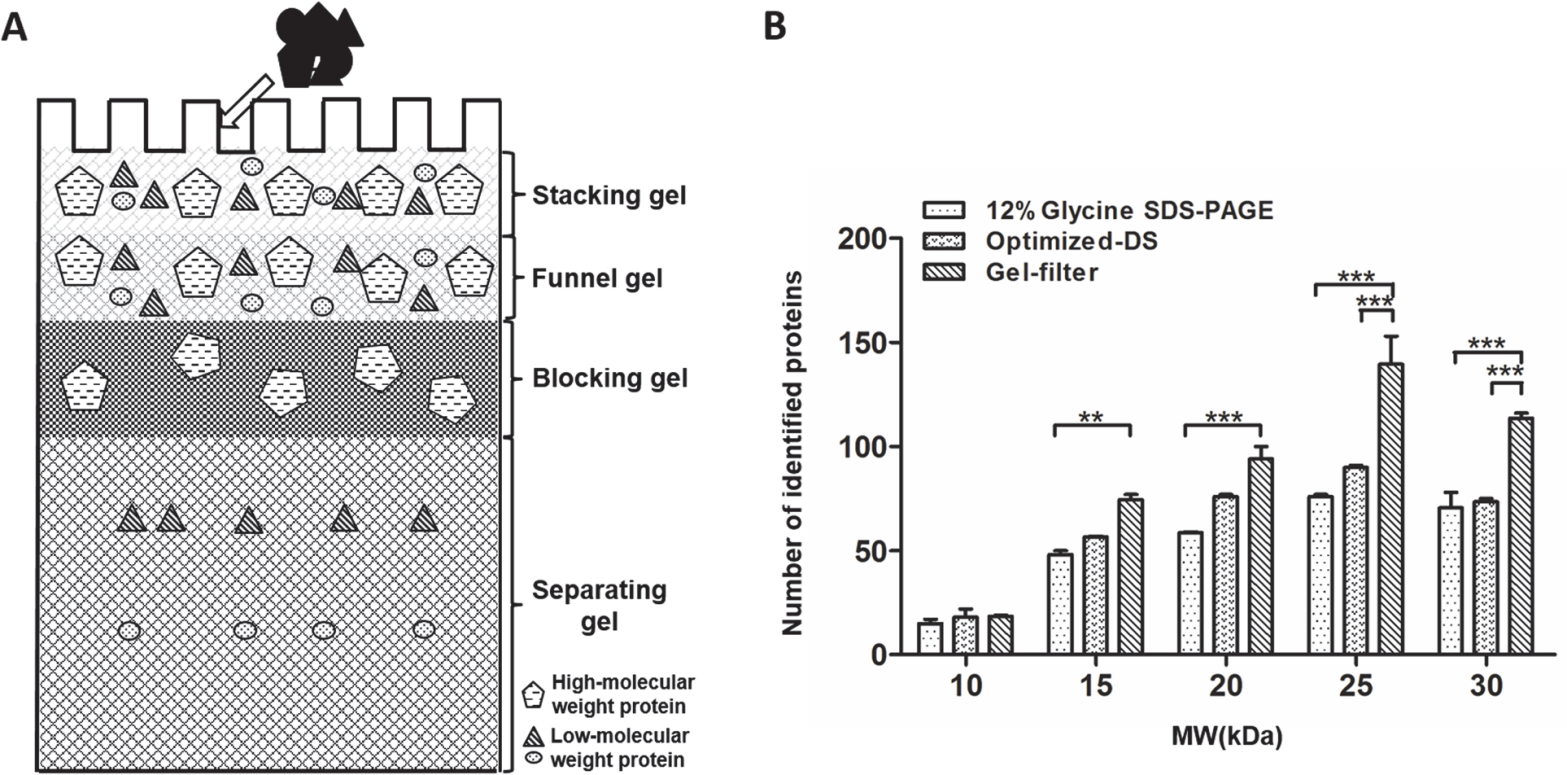
Schematic diagram of gel-filter method and molecular weight distribution of identified LMW proteins from different methods. A. Schematic diagram for the design of four-layer gel-filter method. B. Molecular weight distribution of identified LMW proteins from the three comparison methods.

In this study, we developed a four-layer gel-filter method to block highly abundant HMW proteins and enrich low-abundance and LMW proteins (Fig. 1A). In order to determine the recovery rate of LMW proteins after the process of different approaches, we performed LC-MS analysis by spike-in isotope-labeled protein. We could confidently identify 559 LMW proteins from 10 μL of serum processed with gel-filter method, which was about 67.3% more LMW proteins than that from the regular SDS-PAGE approach. Interestingly, the number of identified LMW proteins from the gel-filter method was also 39.9% more than that from the optimized-DS method. In each molecular weight range bin, the number of identified proteins for gel-filter methods was the highest within three methods (Fig. 1B). More importantly, the quantitative proteomics study revealed that about 33.0% of targeted proteins could be recovered from the gel-filter process, which was about two fold or four fold higher than that in optimized-DS and ProteoMiner approaches, respectively.

## Materials and Methods

### Ethics Statement

The use of human serum samples for this study was reviewed and approved by the Institute of Radiation Medicine at Academy of Military Medical Sciences and Beijing Proteomics Research Center. All participants provided written informed consent (as outlined in PLOS consent form) to participate in this study.

### Materials

All chemical reagents were purchased from Sigma (St. Louis, MO, USA) with analytical grade except formic acid and acetonitrile, which was high performance liquid chromatography (HPLC) grade. Trypsin was purchased from Promega (Madison, WI, USA). ProteoMiner Protein Enrichment Small-Capacity kit was purchased from Bio-Rad (Hercules, CA, USA).

Human serum was obtained from six healthy volunteers (three males and three females; range from 38–80 years old, mean 61.5 ± 14.2 years) to avoid the individual variation from these serum samples. Blood was allowed to clot for 30 min at room temperature, and was then centrifuged at 1,000 × g for 15 min at room temperature to collect serum. Then, the serum from each individual donor was pooled together to save as a single sample for evaluating the features of different processing methods used in this study. The protein concentration of the pooled sample was 65 μg/μL as determined by Bradford protein assay.

### Regular glycine SDS-PAGE and tricine SDS-PAGE Analysis

The regular glycine SDS-PAGE and tricine SDS-PAGE preparation were described as in standard protocols [28,29].

For electrophoresis analysis, the 1 μL of serum sample containing 65 μg proteins was diluted 1:2 ratio with 2 μL of denaturing solution (8 M urea, 10 mM IAA). The sample was then incubated at room temperature for 30 min before centrifugation at 14,000 × g for 5 min. The supernatant was diluted 4:1 ratio with SDS-PAGE loading buffer (1 M Tris-HCl, pH6.8, 10% SDS, 50% glycerol, and 1% bromphenol blue). After mixing and centrifugation at 13,800 × g for 5 min, the supernatant was transferred to a new tube and was ready for gel electrophoresis.

For regular glycine SDS-PAGE analysis, electrophoresis was carried out at a constant 80 V for 3 hours (PowerPac HC, Bio-Rad). Then the gel was briefly rinsed with ultrapure water and stained with Coomassie brilliant blue G-250. After destaining, gels were scanned with a Scanjet image system (HP Scanjet G4050). Gel images were analyzed using Scion Image (http://rsb.info.nih.gov/nihimage/).

For tricine SDS-PAGE analysis, the prepared samples were separated at a constant 30 V until they completely entered into the separating gel from the stacking gel. Then a constant 200 V was maintained until the tracking dye reached the bottom of the gel. The staining, destaining, and image analysis were performed as described above.

### Gel-filter method preparation for LMW protein enrichment

The same glycine SDS-PAGE cassette was used for gel-filter cartridge preparation. From bottom to top, the gel-filter method was composed of a separating gel, blocking gel, funnel gel, and stacking gel. The concentration of separating gel was generally 10–12%. The concentration of blocking gel was 16–20%, which could be adjusted based on the size of objective proteins need to be removed. The concentration of funnel gel was 8–10%, and that for stacking gel was the same as in the standard glycine SDS-PAGE gel method.

The serum sample preparation and electrophoresis parameters for analysis were the same as described in tricine SDS-PAGE analysis.

### Optimized-differential solubilization (Optimized-DS) method

Sample was prepared for the DS method in a manner similar to that described by Kawashima *et al*. [30] with slight modifications. Briefly, 10 μL of serum was diluted 1:2 ratio with 20 μL of denaturing solution (8 M urea, 5 mM DTT) at room temperature avoiding light for 30 min, then alkylated by 20 mM IAA. After addition of 70 μL of ultrapure water, the entire sample was slowly added drop-wise into 1.2 mL of ice-cold acetone, and immediately incubated at -20°C for 4 h, after which it was centrifuged at 5,000 × g for 15 min at room temperature. The precipitate was resuspended in 200 μL of 70% ACN containing 12 mM hydrochloric acid and mixed at 4°C for 1 h to get the LMW proteins. The dissolved LMW protein sample was then centrifuged again at 19,000 × g for 15 min at 4°C. The proteins were lyophilized before analyzed by 12% tricine SDS-PAGE as described above.

### Sample preparation for ProteoMiner

The serum sample was treated with combinatorial peptide ligand library (CPLL) provided by the ProteoMiner kit according to the manufacturer’s protocol. Briefly, the column was washed three times with PBS buffer. Then 200 μL of human serum containing 13 mg proteins was added to the column and incubated at room temperature for 2 h with gentle shaking. The proteins bound on the beads containing combinatorial peptide ligand were washed three times with PBS buffer before eluting with 20 μL of elution buffer (8 M urea, 2% CHAPS) for three times. The eluted proteins were pooled together for further experiments.

For electrophoresis analysis, one twentieth of the eluted proteins from the 200 μL of starting serum sample was mixed with SDS-PAGE loading buffer and then analyzed by 12% tricine SDS-PAGE.

### Protein digestion and peptide identification

After gel electrophoresis, the specific regions in gel lanes were sliced into nine bands based on molecular weight markers. In-gel digestion, peptide extraction and desalting were performed as described previously [31–33]. The eluted peptides were dried again and dissolved with sample loading buffer (1% ACN and 1% Formic acid in water) for MS analysis. The peptide identification was performed on a hybrid LTQ-Orbitrap Velos mass spectrometer (Thermo Fisher Scientific, San Jose, CA) equipped with a Waters nanoACQUITY ultra-performance liquid chromatography (UPLC) system (Waters, Milford, MA) as described previously[33].

All of the three sample processing experiments were repeated three times with the same pooled serum sample to evaluate their reproducibility except for the specific description.

### Determination of recovery of target protein by SILAC-AQUA approach

The protein of Glutathione-S-transferase (GST) was expressed from pGEX-4T vector in the light and heavy SILAC labeled *E*.*coli* BL21 (DE3) strain as described [34]. After completely labeling with [^13^C_6_] lysine (+6.0201 Da), GST protein was purified using gluthatione-Sepharose beads (Qiagen, Valencia, CA) according to the manufacturer instruction. The amount of purified GST was measured by standard BCA protein assay kit (Thermo Scientific, Rockford, IL) and by a Coomassie stained SDS gel as described [35].

The heavy-labeled GST was digested with trypsin and analyzed through LC-MS/MS to get the most appreciated peptides for quantification. Certain amount of human serum samples were spiked with 0.3 μg of light isotope-labeled GST protein and then processed through the treatments of gel-filter, optimized-DS or ProteoMiner approaches as described above. To estimate the protein recovery for each individual process, the treated samples were digested with trypsin and then mixed with the same amount of heavy isotope labeled and tryptic GST peptides followed by nano-LC-MS/MS analysis. The same amount of heavy labeled GST peptides were used as internal standards to evaluate peptide recovery. Detailed quantification method was described as previously [34].

### Data processing and bioinformatics analysis

The raw data were processed with msconvert (ProteoWizard suite version 2.1.2132) using default parameters. The acquired extensible markup language (XML)[36] formatted MS raw data (mzXML) were searched using the Sorcerer-SEQUEST (version 4.0.4 build, Sage-N Research, Inc.) search engine. Data were searched against the NCBI *Homo sapiens* protein database (version 11232012, 35,814 protein sequences) added with 112 frequently observed contaminants (ftp://ftp.thegpm.org/fasta/cRAP/crap.fasta) and decoy sequences derived by peptide-level reversal of all 35,926 target sequences [37]. Search parameters consisted of semitryptic restriction, fixed modification of Cys (+57.0215 Da, alkylation by iodoacetamide), and variable modification of oxidized Met (+15.9949 Da). Peptides with up to two missed cleavages were allowed. Mass tolerance was set to 20 ppm. Only *b* and *y* ions were considered during the database search.

Peptide matches were filtered by a minimal peptide length of 6 amino acids, then grouped by trypticity (only accepted fully and partially tryptic peptides) and charge states [37]. To keep a high quality dataset for comparison, the peptide matches were further filtered by dynamically increasing XCorr and ΔCn cutoffs until the global protein false discovery rate was lower than 1% in each group [38]. We kept the identified proteins with one hit wonder in our list after stringent manual checking due to the limited number of sequenceable peptides resulting from digestion of the LMW proteins.

Because different databases were used in different studies, protein ID of each datasets was converted to gene symbol ID on Uniprot web (http://www.uniprot.org/). Dataset comparison was applied on Venny web (http://bioinfogp.cnb.csic.es/tools/venny/). Protein abundance was normalized as described [39]. MS/MS spectra of peptides were extracted with p-Label [40].

ANOVA was used to determine the statistical significance of data analysis for molecular weight distribution of identified LMW proteins and protein recovery rate. All calculations were performed with Graphpad Prism software system (GraphPad San Diego, CA, USA).

## Results and Discussion

### Development of a novel gel-filter method for the enrichment of LMW proteins

The human serum proteome has been extensively studied to identify protein and peptide biomarkers. However, HAPs with relative HMW in serum make the identification of low abundance LMW proteins through traditional approaches extremely hard. In order to retain HMW proteins and enrich for more LMW proteins for proteomics analyses, we applied a three-layer gel method as part A in S1 Fig., which was similar as described by Schagger *et al*. [28] In this gel method, 5% stacking gel, 16% blocking gel and 10–12% of separating gel were included from top to bottom, respectively. When testing the gel, we found that the loaded amount of the serum sample was still limited because the HAPs with HMW passed through blocking gel and damaged separating gel (Part B in S1 Fig.).

**Fig 2:**
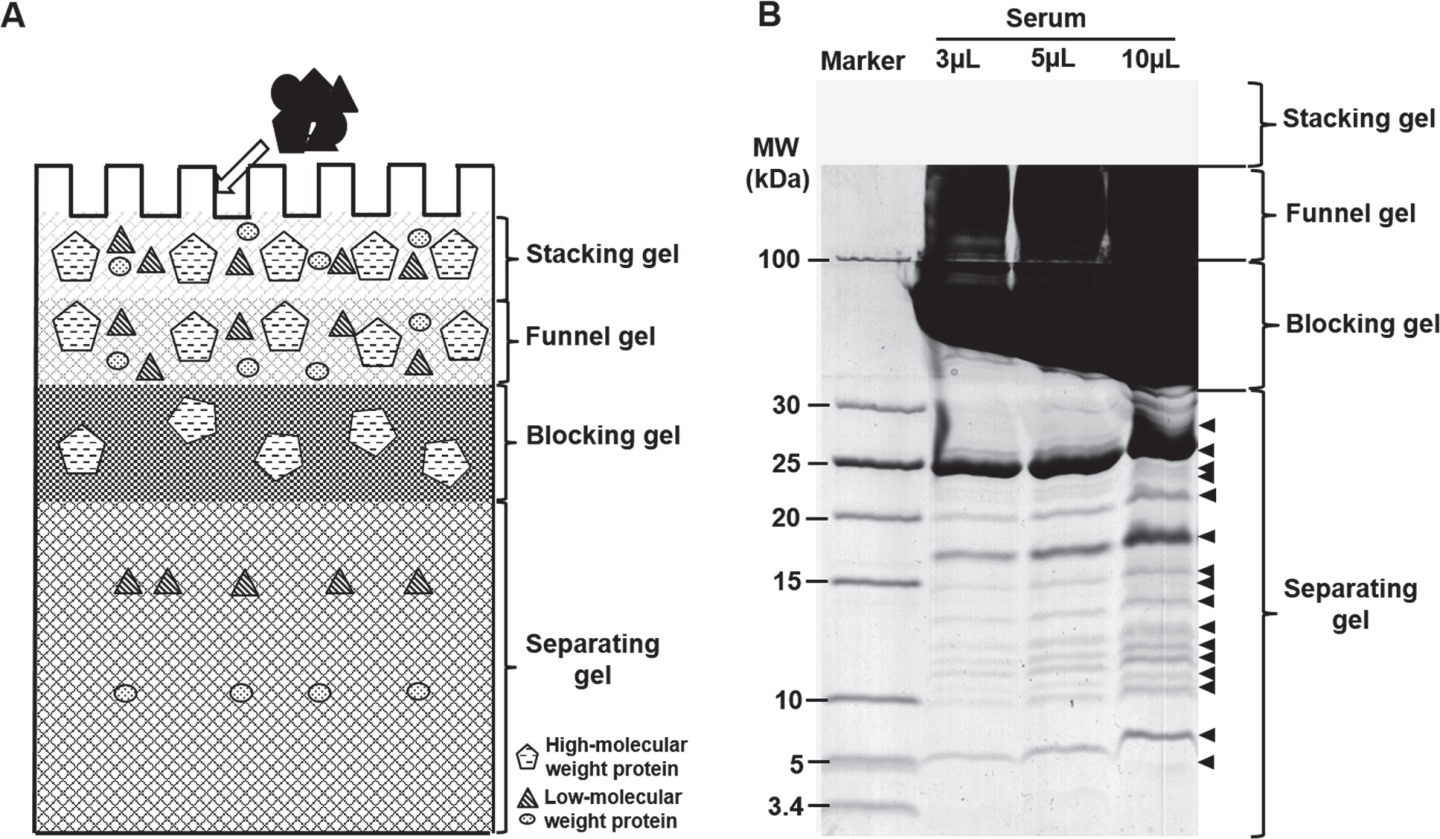
Development of gel-filter method for LMW proteins. A. Schematic diagram of the newly developed gel-filter method. B. The enrichment of LMW proteins from serum through the gel-filter method. The visible bands were labeled with arrows.

In order to completely block the HMW proteins, we further increased the concentration of blocking gel to 20%. However, when the concentrated proteins passed from the stacking gel into the blocking gel, they spread along the interface of stacking gel and blocking gel due to the force of the fast ions from the electrophoresis system and the back pressure from the small pores of the high concentration of the blocking gel (Part C in S1 Fig.). To circumvent this problem, one layer of funnel gel was added between the stacking gel and the blocking gels to hold the concentrated proteins without too much pressure. Thus, a four-layer gel method based on tricine SDS-PAGE was developed and termed the “gel-filter” method (Fig. 2A).

In this “gel-filter” method, proteins are concentrated and focused at the interface between the stacking gel and the funnel gels under the force generated by the fast ions behind the proteins pursuing the slower ions migrating ahead of the proteins. After the proteins pass through the interface and enter the short funnel gel, the proteins will not be separated too much by the molecular sieve of the funnel gel because of its relatively low concentration and short distance of this gel slot, such that this gel serves as a buffer area. When the proteins enter the blocking gel, the HMW proteins are retained due to the back pressure resulting from the extremely high concentration of the gel; however, the LMW proteins can pass through the small sieve of the gel. Because the blocking gel is as short as the funnel gel, the small molecular weight proteins will not be extensively separated. However, these small proteins can be resolved reasonably well after they enter the separating gel. This method utilizes four layers of gel with varied concentrations of acrylamide and bis-acrylamide for the facile enrichment of LMW proteins without any loss during sample preparation. The proteins or peptides in the separating gel can be eluted or extracted out after trypsin digestion for MS analysis.

To test the loaded capacity of the new developed gel-filter method, we increased volumes of serum sample, ranging from 3 μL to 5 μL and 10 μL which containing 195 μg, 325 μg and 650 μg protein, respectively (Fig. 2B). We found that the LMW proteins could be separated from the HMW proteins and resolved very well with multiple sharp and distinguishable protein bands. The density of these protein bands increased with the increase of sample amount, suggesting the enrichment capacity of this gel-filter method for LMW and low abundance proteins. However, when the serum volume was increased to 10 μL which containing 650 μg protein, the leading edge of the HMW proteins began to reach the interface between the blocking gel and separating gel, suggesting the saturation of the loaded amount of this gel method. Therefore, we concluded that the limit of serum amount on this gel-filter method was about 650 μg. In addition, to determine the reproducibility of gel-filter method, two completely repeat experiments were carried out. The intensities of the protein bands and the pattern with the same loaded amount were totally identical. It indicated that the gel-filter method performed with great reproducibility.

### Gel-filter method provided more distinguishable LMW protein bands than regular glycine SDS-PAGE and optimized-DS methods

**Fig 3:**
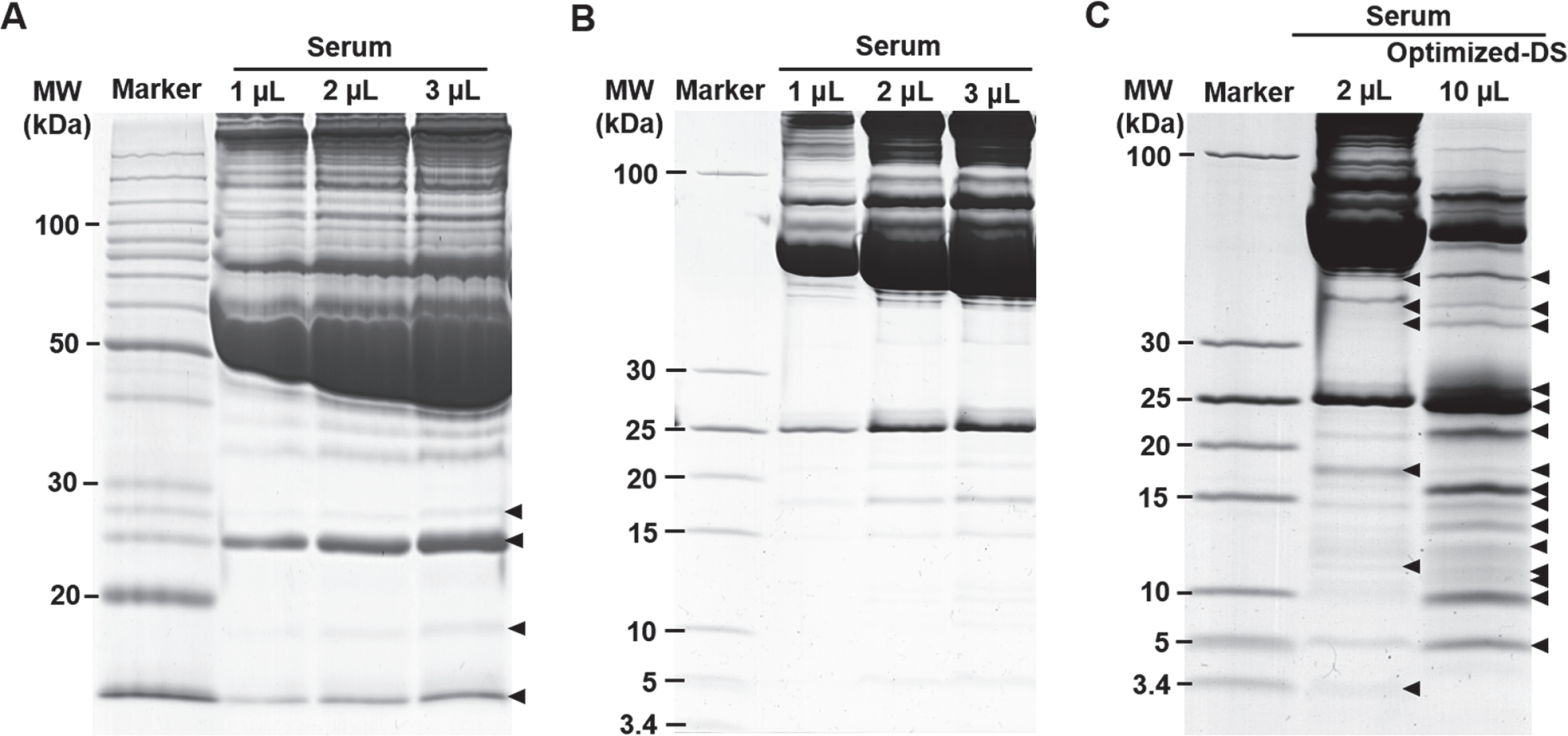
Performance comparison of the enrichment for LMW proteins from human serum using varied methods. A. The human serum was resolved by 12% glycine SDS-PAGE. B. The human serum was resolved by 12% tricine SDS-PAGE. C. The enrichment of human serum LMW proteins after the process of the optimized-DS method. The volume of serum was used as indicated on the figure.

To further evaluate the enrichment efficiency of LMW proteins, three other approaches were applied in comparison with gel-filter method in this study as shown in Fig. 3A. Glycine SDS-PAGE is widely used as a pre-fractionation strategy in proteomics research, which is the preferred electrophoretic method for the separation of proteins in the mass range of 1–500 kDa. However, the small proteins on the leading edge of the gel usually disappeared or heavily smeared [28]. As shown in Fig. 3A, this gel method showed low resolution in LMW range with only four distinguishable bands. A comparison of lanes 2–4 revealed no obvious enhancement on the recovery for LMW proteins with increasing loaded volume of serum sample. Even worse, the loaded amount would also be limited because the high abundant proteins might damage the gel during the electrophoresis.

Tricine SDS-PAGE is an efficient electrophoretic method for resolving proteins smaller than 30 kDa with higher resolution in the LMW range than regular glycine SDS-PAGE [28,41]. As the same for regular glycine SDS-PAGE, there was no visible accumulation of the LMW proteins presenting on the gel with either 1 μL or 3 μL of serum samples (Fig. 3B). However, when the loaded amount was increased to 10 μL, we found that those HAPs were easily overloaded as well, resulting the damage of the gel. This interfered the detectability for diverse but low abundant and LMW proteins with physiological and diagnostic importance (data not shown).

To remove the HAPs from the serum sample and enrich these diverse but low abundant and LMW proteins for MS analyses, various strategies and techniques have been developed in the past decade [8,16–22]. Among them, the differential solubilization method recently developed by Kawashima *et al*. has been selected for comparison because of its high efficiency and reproducibility for extracting LMW proteins [30]. We applied this method, and did see much better performance on the enrichment of low abundant proteins in serum sample because the abundant HMW proteins were significantly reduced. By this way, we could detect about ten enriched LMW protein bands below 30 kDa molecular weight range (S2 Fig.), which was consistent with Kawashima’s result. In order to increase the efficiency of this DS method, we slightly modified the Kawashima’s method by adding certain volume of ultrapure water to the diluted and denatured serum sample as described above to reduce the protein concentration. Then the diluted sample was slowly dropped into 1.2 mL ice-cold acetone, and immediately incubated at -20°C for 4 h. This helped to slow down the precipitation process for proteins mixed with acetone. By following this optimized procedure (optimized-DS), we could detect about twelve bands below 30 kDa molecular weight range (Fig. 3C, as indicated as arrows). However, when we compared the pattern between lanes for regular sample preparation and optimized-DS methods, we found that the intensity of the arrowhead-marked bands in the treated sample was not positively correlated with those in the untreated serum. This might be caused by partial solubilization of some potentially important proteins, which resulting their existing in both of the soluable and insoluable fractions [9]. Even worse, some dominant or distinguishable LMW protein bands from original serum might disappear after DS treatment. This result indicated that the original abundance of proteins in the serum sample was changed after the DS treatment. Hence, the results from the DS treated serum may not be able to reflect the original nature of the sample (Fig. 3C lane3) and bother the quantitative comparison between the physiological and pathological samples for biomarker screening. Therefore, the remaining unsatisfactory of the enrichment methodology for LMW proteins requires the development of unbiased method with even higher efficiency.

Compared with the optimized-DS, the gel-filter method here performed better efficiency on the enrichment of LMW proteins. When we loaded 3 μL of serum sample, we could clearly detect 12 sharp bands in the 5–30 kDa molecular weight range, although some bands were thin because of low amount of protein (Fig. 2B). The number of protein bands from 3 μL of serum was the same as what we got from 10 μL of same sample treated with optimized-DS method, which was the best result we got from the DS method. When the serum sample volume was increased to 5 μL, the number of detectable protein bands between 3.4 kDa and 30 kDa molecular weight range even increased to 15, which was 25% more than what we got from the optimized-DS method. Further analysis of 10 μL of serum sample increased the proportion of LMW proteins, but the number of total protein bands remained the same (Fig. 2B). These results indicated that the sample loaded capacity, enrichment efficiency and detection sensitivity of the gel-filter method were better than all of the methods we compared here. More importantly, we did not notice any pattern change on protein bands compared with both of the regular SDS-PAGE and tricine SDS-PAGE results, which remained the original features of protein components in the serum. This is important in quantitative proteomics study.

### Evaluation of the enrichment efficiency for LMW proteins through multiple approaches by LC-MS/MS analysis

In order to further evaluate the efficiency of these three methods for LMW protein enrichment in this study, the gel bands lower than 30 kDa were digested with trypsin and then analyzed by LC-MS/MS twice. A total number of 334 proteins was identified from 1 μL serum sample separated by regular 12% glycine SDS-PAGE across two replicate runs (Part A in S3 Fig.). We also tested 3 μL serum sample separated by the same method. However, there was almost no changes on the number of identified proteins, suggesting the saturation of the separation power on this regular glycine SDS-PAGE method (data not shown). For optimized-DS method, we totally identified 400 LMW proteins from two replicate runs (Part B in S3 Fig.). As expected, the number of identified protein from optimized-DS method was slightly higher than that from the regular glycine SDS-PAGE as we saw more separated protein bands on optimized-DS method. The overlapped proteins between these two methods were 154, representing 46.1% and 38.5% of the identified proteins from glycine SDS-PAGE and optimized-DS methods, respectively. However, we identified 559 LMW proteins from the same serum sample treated with our newly developed gel-filter method with same sample amount as in optimized-DS method (Part C in S3 Fig.), which was about 67.4% more than we got from the conventional glycine SDS-PAGE and 39.8% more than that from the optimized-DS method. In addition, the gel-filter approach achieved about 55% overlap of identified proteins between two complete repeat experiments with the same serum sample. However, these for 12% glycine SDS-PAGE and optimized-DS methods were 44% and 48%, respectively (S4 Fig., S1 Table), which was slighter lower that what we got from gel-filter method.

**Fig 4:**
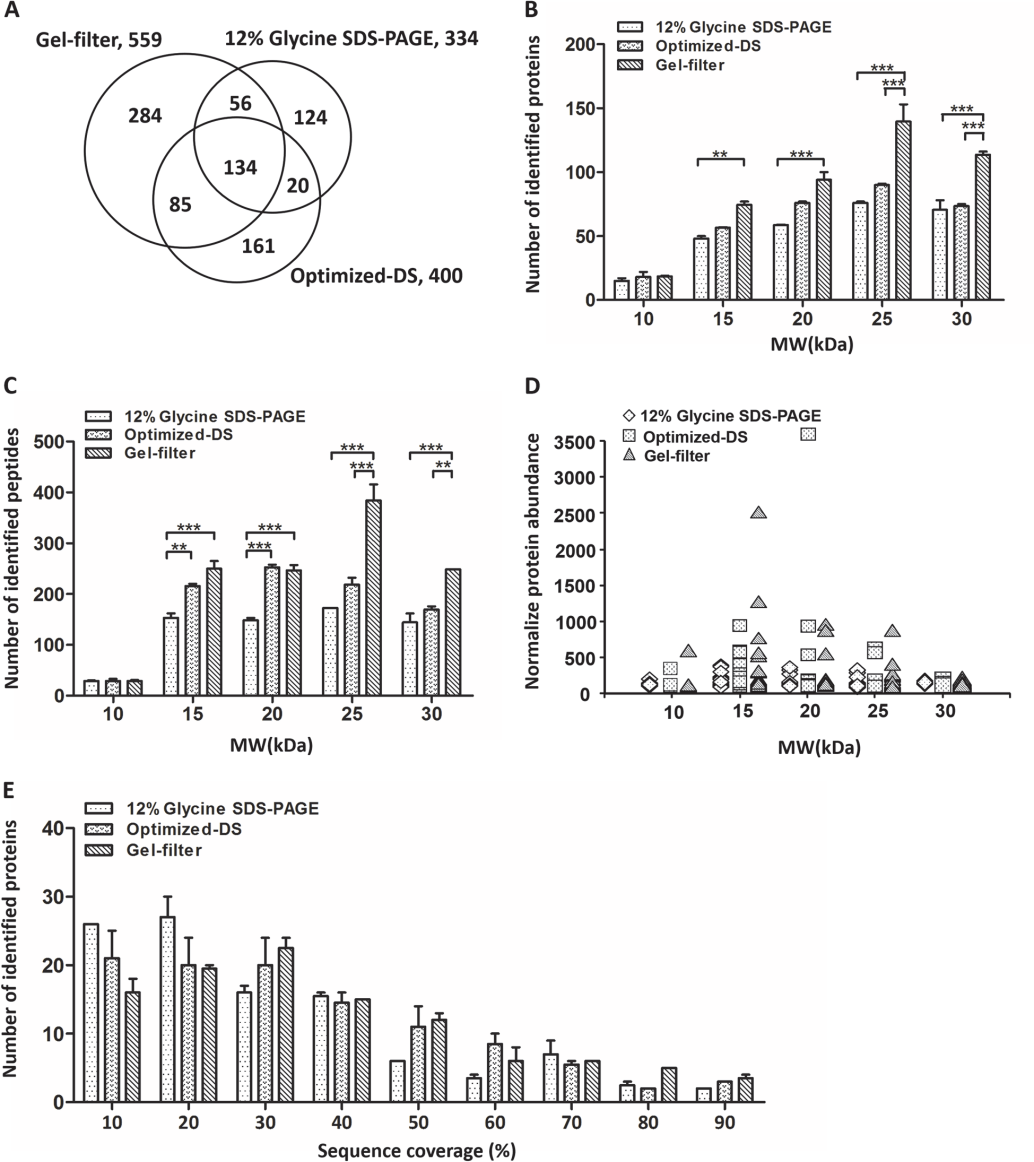
The comparison of identified human serum LMW proteins from the representative methods and gel-filter method. A. Venn diagram for the overlap of identified LMW proteins from the same sample processed by gel-filter, glycine SDS-PAGE and optimized-DS methods. B. Molecular weight distribution of identified LMW proteins from these three approaches. C. The distribution of identified peptides from the LMW proteins after the process of these three approaches. D. Abundance analysis of commonly identified 134 LMW proteins from these three strategies. E. Sequence coverage distribution of the commonly identified LMW proteins from these three methods.(*:P < 0.05, **:P < 0.01)

Totally we identified 864 LMW proteins from these serum samples treated with three methods. Comparing these three datasets, we found that 134 proteins were commonly identified, representing about 15.5% of these 864 LMW proteins identified (Fig. 4A). A total of 190 proteins was commonly identified by both the gel-filter and regular glycine SDS-PAGE methods, which occupying 57% of all identified proteins from glycine SDS-PAGE. This was consistent with the same basic principle of these two methods. We also had 219 proteins commonly identified by the gel-filter and optimized-DS methods, representing 54.8% of the total of 400 proteins identified using the optimized-DS method. The percentage for shared proteins on both of the optimized-DS and regular glycine SDS-PAGE methods were even lower (46.1% and 38.5%, respectively). This was also consistent with our gel imaging results because some bands disappeared in the optimized-DS process compared with those for the regular glycine SDS-PAGE and gel-filter methods.

To test for bias of these methods with respect to enrichment of LMW proteins, we categorized the identified proteins based on their molecular weight. As indicated in Fig. 4B, the patterns of these three methods were pretty similar. Globally, the number of proteins identified with regular glycine SDS-PAGE was the lowest one in each molecular weight range bin. It also appeared that the number of identified proteins from the regular glycine SDS-PAGE increased over the 10–25 kDa molecular weight range, and kept stable between 25–30 kDa bins. It suggested that this method does not have an obvious enrichment effect on LMW proteins because proteins with higher molecular weight were more easily identified due to the higher number of tryptic peptide. However, the number of proteins identified between 10 kDa and 25 kDa with the optimized-DS method was higher although the number of identified proteins with molecular weight range between 25 kDa and 30 kDa decreased. This was consistent with the principle that the DS method has an enrichment effect on LMW proteins. As the same, we found that the molecular weight distribution of the proteins identified with the gel-filter method also looked like a parabolic curve, in which the highest number centered at about 25 kDa. In each molecular weight range bin, the number of identified proteins for gel-filter method was the highest within these three methods.

A similar trend was observed regarding the distribution of identified peptides (Fig. 4C). There was significant difference on the number of identified proteins/peptides between gel-filter and glycine SDS-PAGE methods (*p* <0.001) in the molecular weight range of 15–30 kDa. However, the significant difference on the number of identified proteins/peptides between gel-filter and DS methods was observed only in the molecular weight range of 25–30 kDa. The result further confirmed the higher efficiency of gel-filter method on the enrichment of LMW proteins.

To compare the abundance feature of the identified proteins from different methods, we categorized proteins based on their theoretical molecular weight and relative abundance represented by their spectral counts in mass spectrometry analysis [39]. As expected, the glycine SDS-PAGE performed the narrowest abundance distribution on all of the molecular weight range bins, representing the lowest abundance of identified proteins. This might also be consistent with the fact that this method did not show specific enrichment on LMW proteins. However, the other two strategies showed a similar trend in the abundance change of identified proteins (Fig. 4D).

To further understand the enrichment factor of these methods on LMW proteins, we calculated the percentage sequence coverage for all of the commonly identified proteins. We found that the sequence coverage of proteins obtained using regular glycine SDS-PAGE was lower than that in either DS or gel-filter methods. The number of identified proteins via gel-filter method kept a higher level on all of the sequence coverage from 30–90%, however, a gradual decreased trend in percent coverage was observed with the glycine SDS-PAGE method (Fig. 4E). These results further demonstrated that the LMW proteins can be enriched more efficiently and without any bias by the gel-filter method developed here.

**Fig 5:**
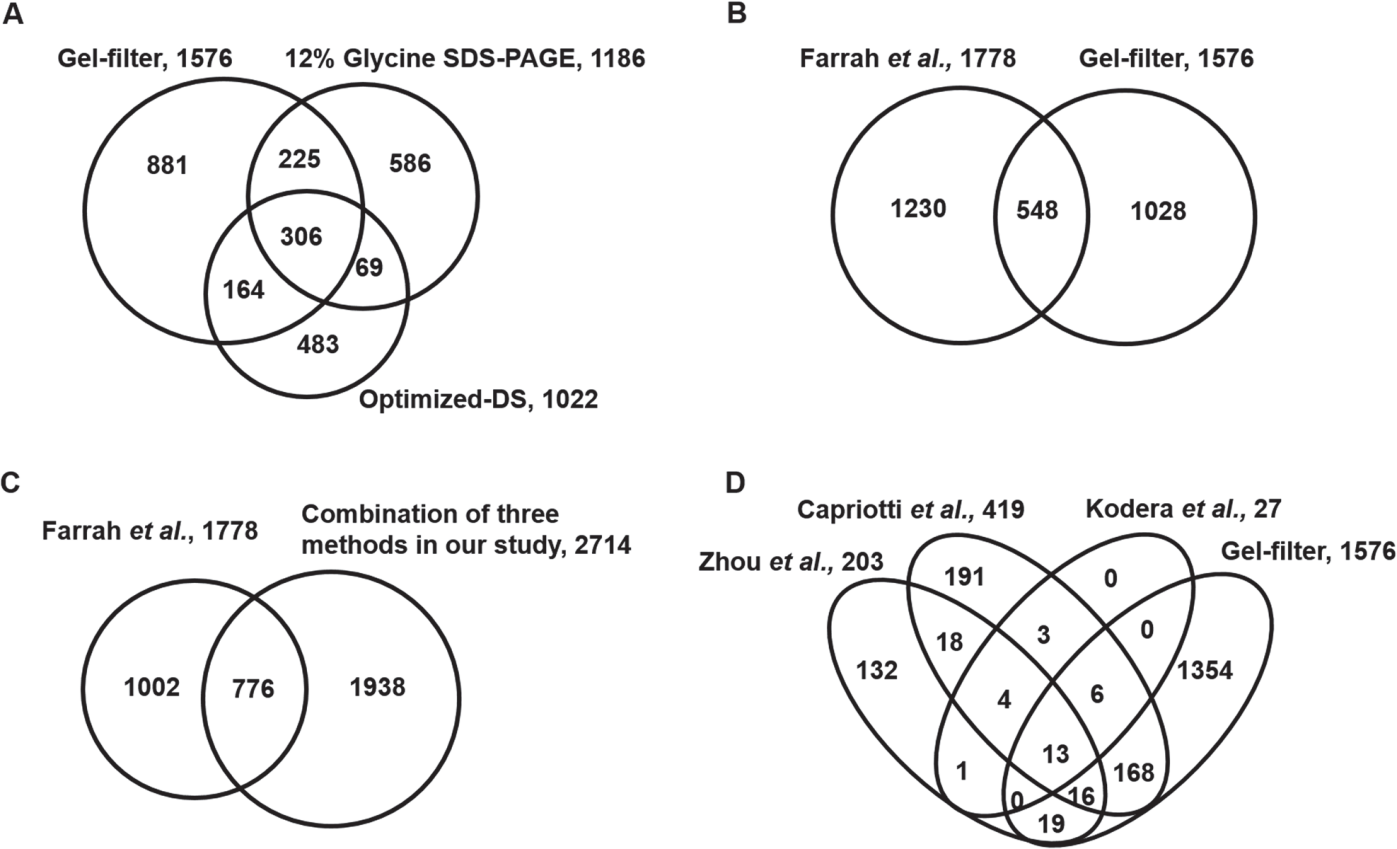
The comparison of identified human serum proteins from gel-filter method and the publically available largest datasets. A. Venn diagram for all of the proteins identified from three methods, including gel-filter, 12% glycine SDS-PAGE and the optimized-DS methods. B. Venn diagram showing the overlap between proteins identified from gel-filter method and the collected datasets for all of the published serum proteomics studies by Farrah et al. C. Venn diagram showing the overlap between proteins identified from all of the three methods used in our study and the collected datasets as in B. D. Venn diagram showing the overlap of proteins identified for gel-filter method and other three serum LMW proteomics studies. All proteins were converted to Gene symbol ID for comparison.

In order to compare the features of these three methods on whole proteome scale, we then analyzed all of the identified proteins. As shown in Fig. 5A, we totally identified 1,576, 1,022 and 1,186 proteins from two repeated runs of gel-filter, optimized-DS and 12%glycine SDS-PAGE methods, respectively (S2 Table). The shared proteins for all of these three datasets were 306, which represented 29.9% of the identification on optimized-DS method and 19.4% of the identified proteins using gel-filter method. The individual percentage for proteins commonly present on the gel-filter and optimized-DS methods and on the glycine SDS-PAGE and the optimized-DS methods was about 30%, which was slightly lower than the 44.8% of overlapped portion between the gel-filter and glycine SDS-PAGE methods. This, again, was consistent with the similar principles of these two gel-based methods and the different principle of the DS method.

The total 1,576 proteins identified from the gel-filter method, providing one of the largest datasets publicly available for serum proteomics study so far from small volume of serum sample in one experiment. This number was just slightly lower than the pooled database containing 1,778 proteins from Farrah’s group for all of the previously published serum proteomics data (Fig. 5B) [42]. The total proteins presenting in these two datasets were 2,806. By comparing our data with the summarized dataset, we found a total of 548 proteins shared on both, represented 34.8% of all of the proteins identified using the gel-filter method and 19.5% of all 2,806 identified proteins, respectively. Similar findings of low overlap between different serum proteomics studies have been reported previously [8,43]. These results suggest that the serum might contain more proteins. Therefore, multiple approaches should be adopted in the serum proteomics study to achieve even higher coverage of the serum proteome.

By combining all of the proteins identified from the same serum sample treated with all of the three methods in this study, we totally identified 2,714 proteins, which was about 52.6% more proteins than the pooled datasets mentioned above. The number of proteins that overlapped between these two datasets was 776, representing 28.6% of all 2,714 proteins we identified and 20.9% of all 3,716 proteins identified from human serum so far (Fig. 5C). We also compared our data with those from three publicly available proteomic datasets specifically examining LMW proteins from serum. Among them, Kawashima *et al*. identified 27 LMW proteins with the DS method they developed. The overlapped proteins between our data and Kawashima’s data was 19. Zhou *et al*. [44] identified 203 proteins by utilizing multiple antibodies to deplete the most abundant proteins in serum. The overlap between Zhou’s data and ours was 48 proteins, representing 23.7% of Zhou’s entire dataset and 3.1% of our dataset. Capriottic *et al*. [45] tried to identify more LMW proteins by combining multiple techniques, including immunoprecipitation, ultracentrifugation and a hydrogel nanoparticles material. Totally they identified a total of 419 proteins, of which 203 were covered by our method as well, representing 48.5% of all 419 proteins identified in their study. However, the overlap between Capriottic’s data and Zhou’s data was only 51 proteins, suggesting that the principles of their enrichment methods differed. A total of 13 proteins was commonly identified by all four of these studies, which represents about 48% of all proteins identified using the DS method (Fig. 5D). Interestingly, 10 of the proteins have a molecular weight ≤ 30 kDa with concentrations ranging from reasonably high (such as apolipoprotein A-I precursor, 1–2 mg/L in serum) to quite low (such as apolipoprotein C-II precursor, 20–60 ng/mL in serum), indicating the potential bias of these methods for the identification of LMW proteins. The largest dataset we obtained from our gel-filter method further emphasized the good efficiency of our method for the identification of low-abundance and LMW proteins from serum samples.

### Higher enrichment efficiency of Gel-filter method revealed by quantitative proteomics

**Fig 6:**
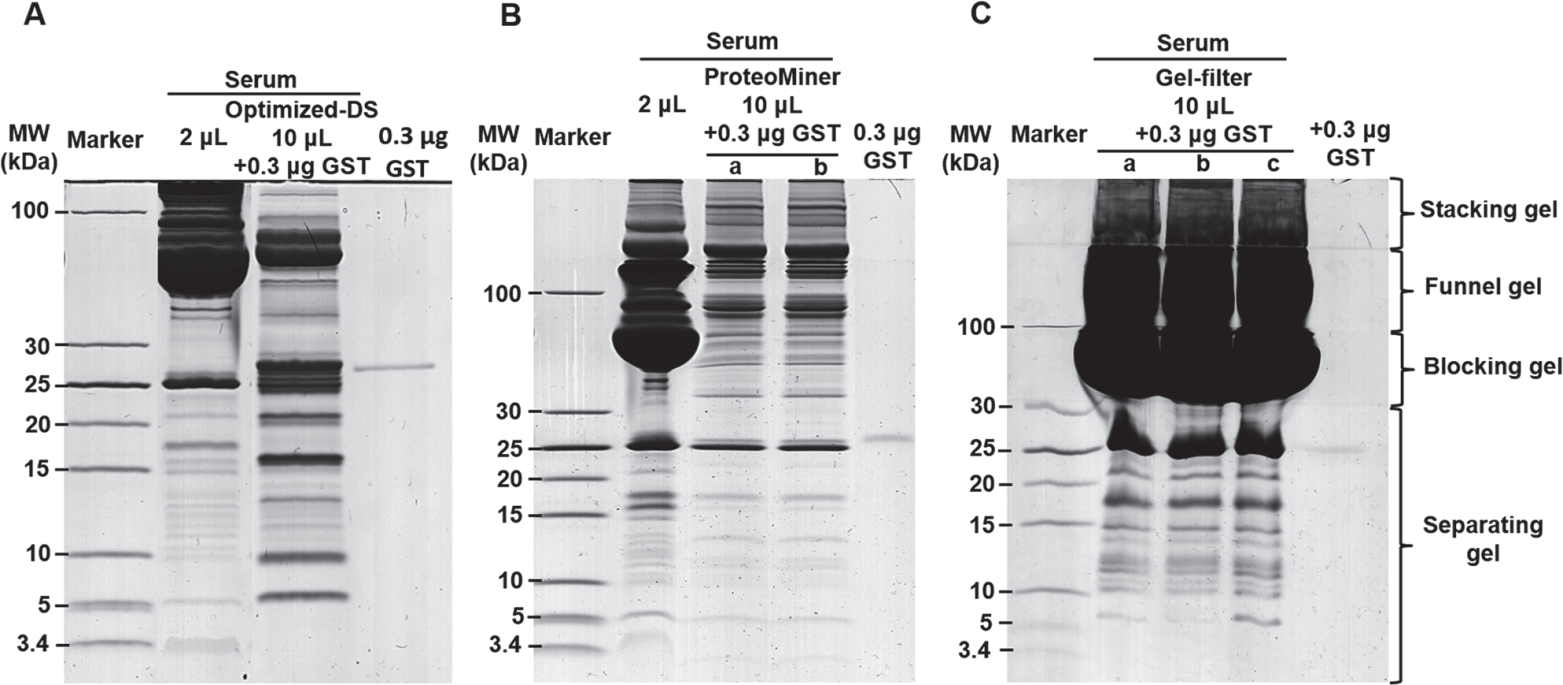
Performance comparison of the recovery rater for LMW proteins from human serum using optimized-DS, ProteoMiner and gel-filter methods. A. Human serum proteome analyzed by the optimized-DS approach. On lane 1, 2 μL of untreated human serum was loaded. On lane 3, 10 μL of human serum was processed with the optimized-DS method after mixed with 0.3 μg regular GST protein. B. The human serum was processed with ProteoMiner method. On lane2, 2 μL of untreated human serum was saved as control as in A. On lane 3 (a) and 4 (b), 5% of eluates from the starting 200 μL of human serum sample treated with ProteoMiner was mixed with 0.1 or 0.3 μg of regular GST proteins and resolved on SDS-PAGE gel. C. Gel-filter analysis of human serum. On lane 2–4 (a-c), 10 μL of human serum samples were mixed with 0.3 μg of regular GST protein and analyzed by gel-filter method. On lane 4 (A) and 5 (B and C), 0.3 μg of regular GST protein alone was loaded as control.

**Fig 7:**
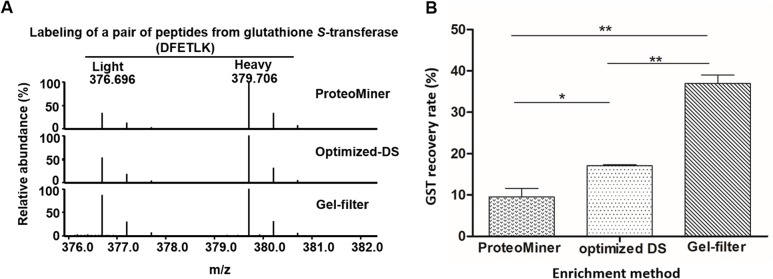
Protein recovery analysis in the process of optimized-DS, ProteoMiner and gel-filter approaches for human serum samples. A. Peptide quantification with SILAC labeled GST as internal standard. Same amount of peptides digested from heavy labeled GST was spiked in the samples processed with optimized-DS, ProteoMiner and gel-filter methods, and then analyzed by LC-MS/MS. B. Histogram of protein recovery after the treatment through these three strategies. Error bars indicate mean ± SD. The whole experiment was repeated twice (*: P < 0.05, **: P < 0.01).

In addition to simple and easy operation, SILAC-AQUA strategy has been confirmed as the accurate quantitative proteomics approach, which has been widely applied in quantitative proteomics studies of various cells, tissues or organisms[46,47]. To determine the recovery rate of proteins during the sample process, we applied a SILAC-AQUA approach with recombinant GST [48]. In order to do that, we expressed and purified light and heavy SILAC labeled GST proteins, respectively. The workflow was presented in S5 Fig. In the case of the optimized-DS and gel-filter methods, 0.3 μg of light labeled GST protein was mixed with 10 μL of serum samples before treatment and gel analysis (Fig. 6 A, C). For ProteoMiner, 6 μg of light labeled GST protein was mixed with 200 μL of serum before sample processing with the kit. As one twentieth of the balanced sample was used for gel analysis, ratio between the GST and the serum was kept the same (Fig. 6B). Then the tryptic peptides from these three treatments were spiked with the same amount of trypsin digested heavy labeled GST before LC-MS/MS analysis. Quantification of peptide ions was performed based on the extracted ion current (XIC) areas (Part A-G in S6 Fig., S3 Table). As shown in Fig. 7 A, B, the gel-filter method could recover 33.1 ± 0.01% (calculated based on two peptides) of spiked light labeled GST protein after the whole sample process. However, the recovery rates for the optimized-DS and ProteoMiner methods were 18.7 ± 0.01% (calculated based on two peptides) and 9.6 ± 0.03% (n = 2), respectively. This recover rate here for ProteoMiner was slightly higher than 3% of recovery rate reported previously[49]. These results further demonstrated that the gel-filter method generally performed higher recovery rate than the other methods we tested.

## Conclusions

We developed a gel-filter method utilizing varied gel concentrations in multiple layers of tricine SDS-PAGE-based gels. The layers include a stacking gel, funnel gel, blocking gel, and separating gel. Upon electrophoresis, proteins are concentrated in stacking gel and completely shifted into a short-length intermediate funnel gel. The LMW proteins pass through the high-concentration blocking gel layer smoothly and are further separated in the separating gel. However, HMW proteins are effectively obstructed. The newly developed gel-filter method is a simple but high-yield method for enriching LMW proteins/peptides in serum without bias. Therefore, the method here are expected to be highly instructive for the LMW proteome study and should play a prominent role in screening for LMW protein biomarkers from serum through proteomic analyses.

## Supporting Information

S1 Data This file contains supporting data. (DOC)

S1 Fig Development of gel-filter method. A. Schematic diagram for the design of three-layer gel-filter method. B. The performance of the developed three-layer gel-filter method for human serum proteome. Lane1, marker; lane 2–3, 3 and 9 μL of human serum samples were resolved on the gel, respectively. C. The performance of the developed three-layer gel-filter method with 20% blocking gel in the middle for human serum proteome. Lane1, marker; lane 2–3, 3 and 9 μL of human serum samples were run on the gel, respectively.
(TIF)

S2 Fig Analysis of human serum proteome with regular DS methods. Lane 1, marker; lane 2, 2 μL of untreated human serum; lane 3, 10 μL of human serum.
(TIF)

S3 Fig Venn diagrams for two technical replicate runs for the enrichment of LMW proteins from human serum. A. 12% Glycine SDS-PAGE, B. Optimized-DS method, C. Gel-filter method.
(TIF)

S4 Fig Venn diagrams for two independent biological experiments for the enrichment of LMW proteins from human serum. A. 12% Glycine SDS-PAGE, B. Optimized-DS method, C. Gel-filter method.
(TIF)

S5 Fig Strategies used for the comparison in this study. The comparison for recovery rate of LMW proteins was performed through three different approaches, including optimized-DS, ProteoMiner and Gel-filter methods.
(TIF)

S6 Fig Protein recovery analysis in the process of optimized-DS, ProteoMiner and gel-filter methods for human serum samples. Peptide quantification was performed by SILAC-AQUA methodology. Same amount of peptides digested from light labeled GST was spiked in the samples processed with optimized-DS, ProteoMiner and gel-filter methods, and then analyzed by LC-MS/MS. A and C. Optimized-DS method; E and G. ProteoMiner; I and K. Gel-filter method. Representative spectra with SILAC pairs of the peptide DFETLK and VDFLSK were selected for quantification as indicated.
(TIF)

S1 Table Protein identification for one independent biological experiment resulting from LC-MS/MS analysis for different method treated serum samples. (XLS)

S2 Table Protein accession number, corresponding total peptide hits and spectral counts resulting from LC-MS/MS analysis for different approaches treated serum sample. (XLS)

S3 Table Quantification of recovery of the spike-in GST protein treated with different approaches. (XLS)
