# Supplementary material for: Development of Gel-Filter Method for High Enrichment of Low-Molecular Weight Proteins from Serum
Source: PLoS One. 2015 Feb 27;10(2):e0115862. doi: 10.1371/journal.pone.0115862 (PMC4344347; doi:10.1371/journal.pone.0115862)
Supplement: S1 Data — (DOC) [file pone.0115862.s001.doc]

**S1 Data.** DOI of each file.

| **File** | **Doi** | **Type** |
| --- | --- | --- |
| Table S1 | 10.6084/m9.figshare.1277843 | Xls |
| Table S2 | 10.6084/m9.figshare.127794 | Xls |
| Table S3 | 10.6084/m9.figshare.1277956 | Xls |
| Figure S1 | 10.6084/m9.figshare.1278089 | Tif |
| Figure S2 | 10.6084/m9.figshare.1278131 | tif |
| Figure S3 | 10.6084/m9.figshare.1278187 | tif |
| Figure S4 | 10.6084/m9.figshare.1278233 | tif |
| Figure S5 | 10.6084/m9.figshare.1278378 | tif |
| Figure S6 | 10.6084/m9.figshare.1278467 | tif |
| Figure 1 | 10.6084/m9.figshare.1279194 | tif |
| Figure 2 | 10.6084/m9.figshare.1279195 | tif |
| Figure 3 | 10.6084/m9.figshare.1279196 | tif |
| Figure 4 | 10.6084/m9.figshare.1279197 | tif |
| Figure 5 | 10.6084/m9.figshare.1279198 | tif |
| Figure 6 | 10.6084/m9.figshare.1279199 | tif |
| Figure 7 | 10.6084/m9.figshare.1279200 | tif |
