# Supplementary figures and images for: Development of Gel-Filter Method for High Enrichment of Low-Molecular Weight Proteins from Serum
Source: PLoS One. 2015 Feb 27;10(2):e0115862. doi: 10.1371/journal.pone.0115862 (PMC4344347; doi:10.1371/journal.pone.0115862)

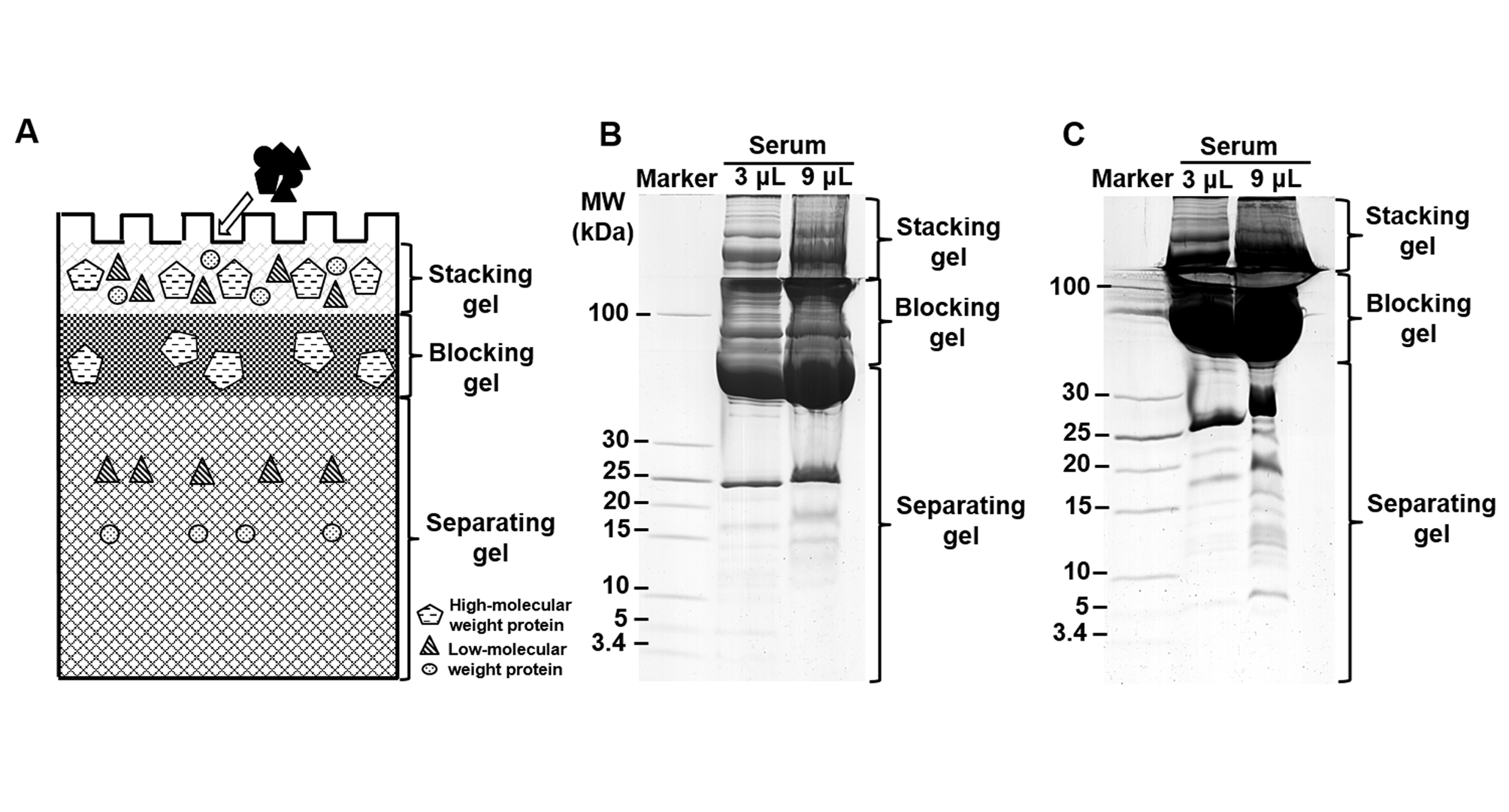

Supplement: S1 Fig — A. Schematic diagram for the design of three-layer gel-filter method. B. The performance of the developed three-layer gel-filter method for human serum proteome. Lane1, marker; lane 2–3, 3 and 9 μL of human serum samples were resolved on the gel, respectively. C. The performance of the developed three-layer gel-filter method with 20% blocking gel in the middle for human serum proteome. Lane1, marker; lane 2–3, 3 and 9 μL of human serum samples were run on the gel, respectively. (TIF) [file pone.0115862.s002.tif]

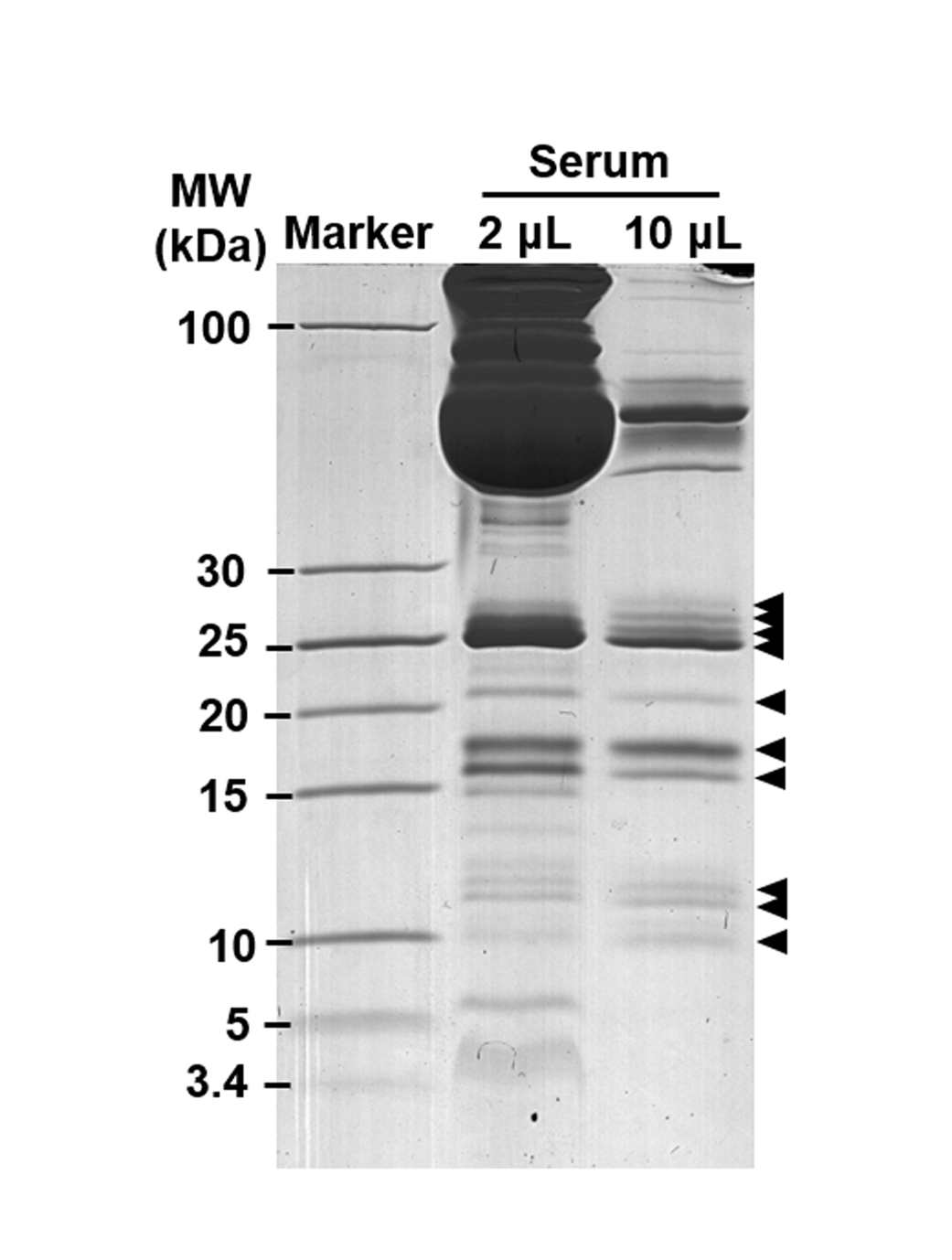

Supplement: S2 Fig — Lane 1, marker; lane 2, 2 μL of untreated human serum; lane 3, 10 μL of human serum. (TIF) [file pone.0115862.s003.tif]

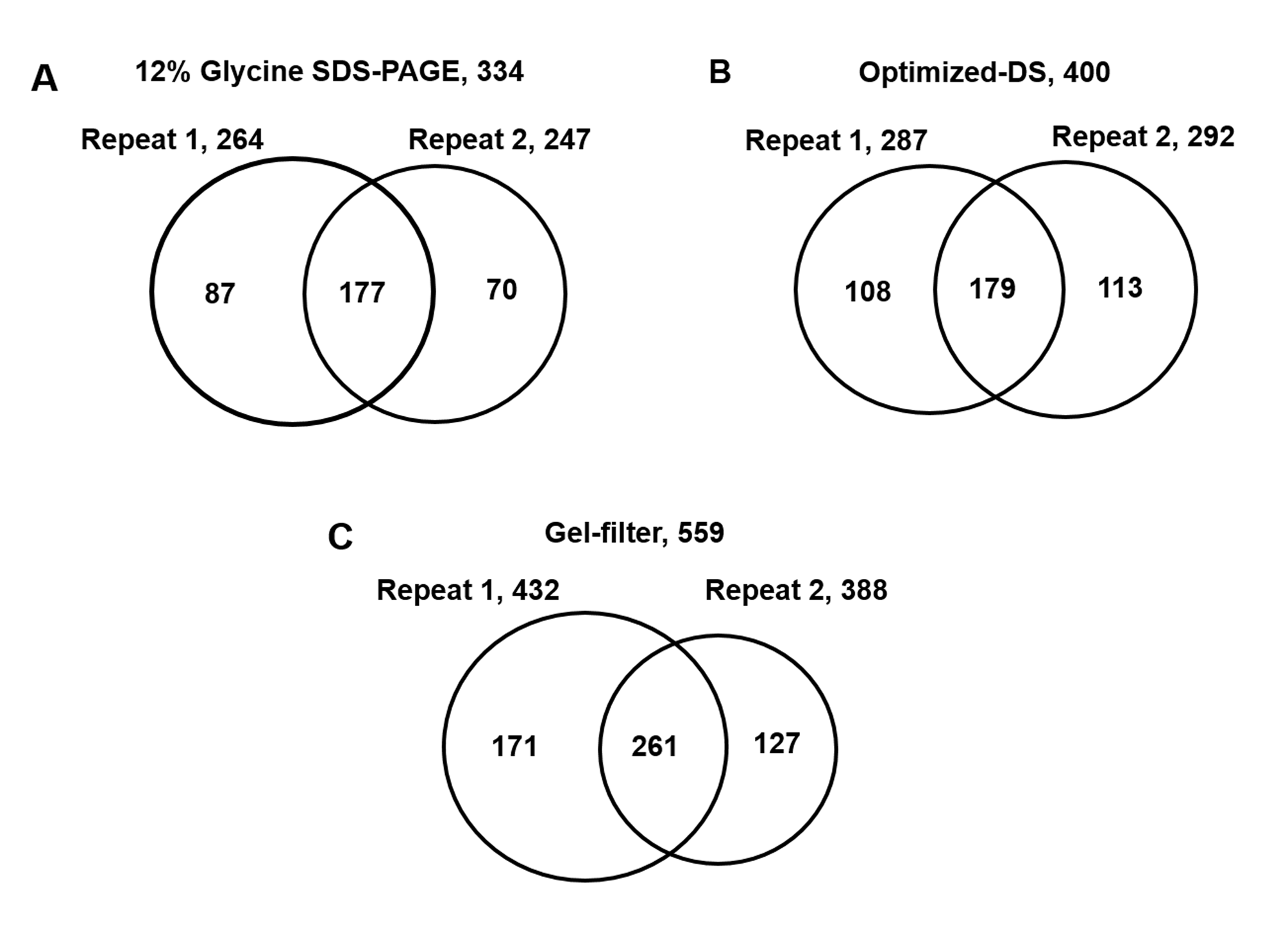

Supplement: S3 Fig — A. 12% Glycine SDS-PAGE, B. Optimized-DS method, C. Gel-filter method. (TIF) [file pone.0115862.s004.tif]

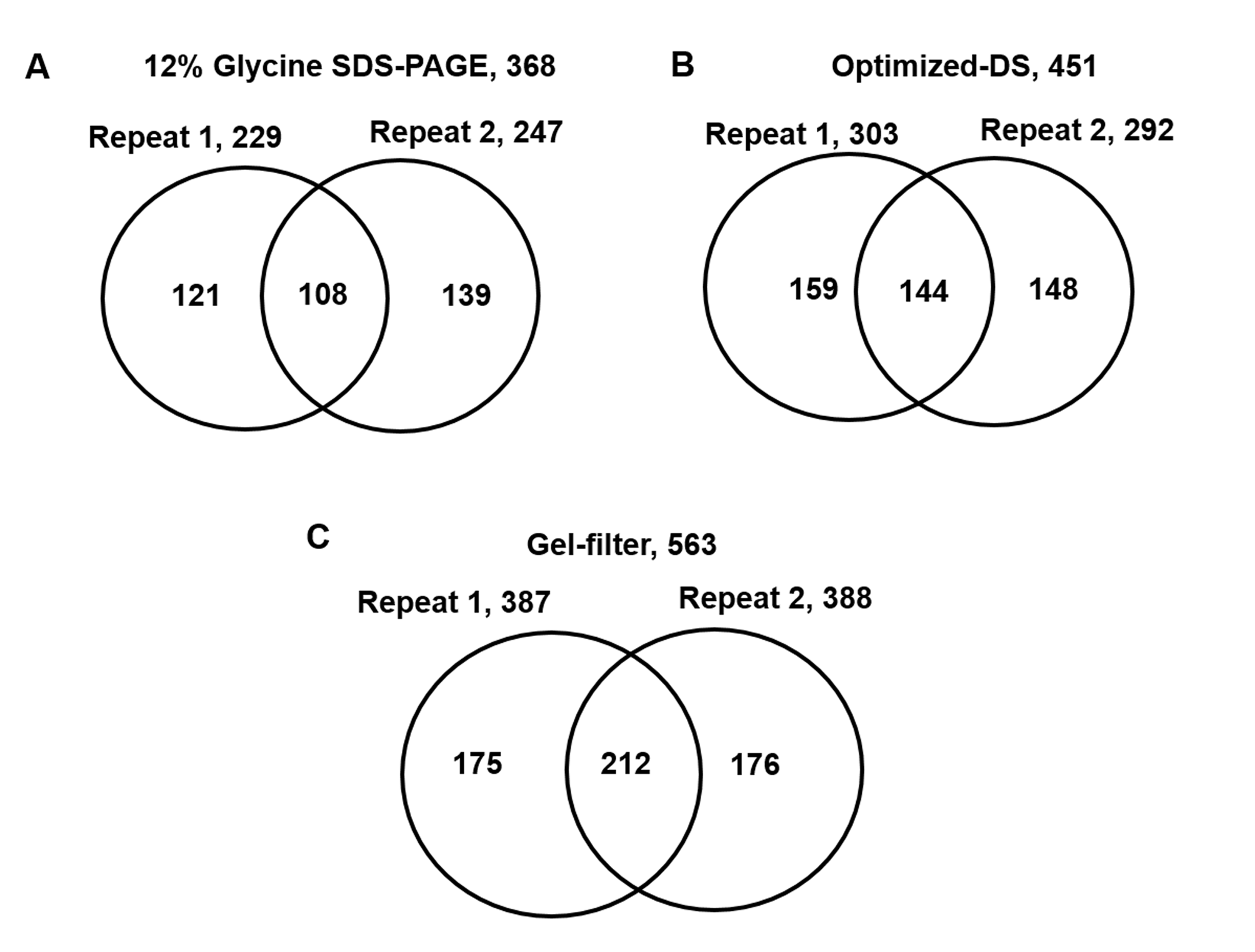

Supplement: S4 Fig — A. 12% Glycine SDS-PAGE, B. Optimized-DS method, C. Gel-filter method. (TIF) [file pone.0115862.s005.tif]

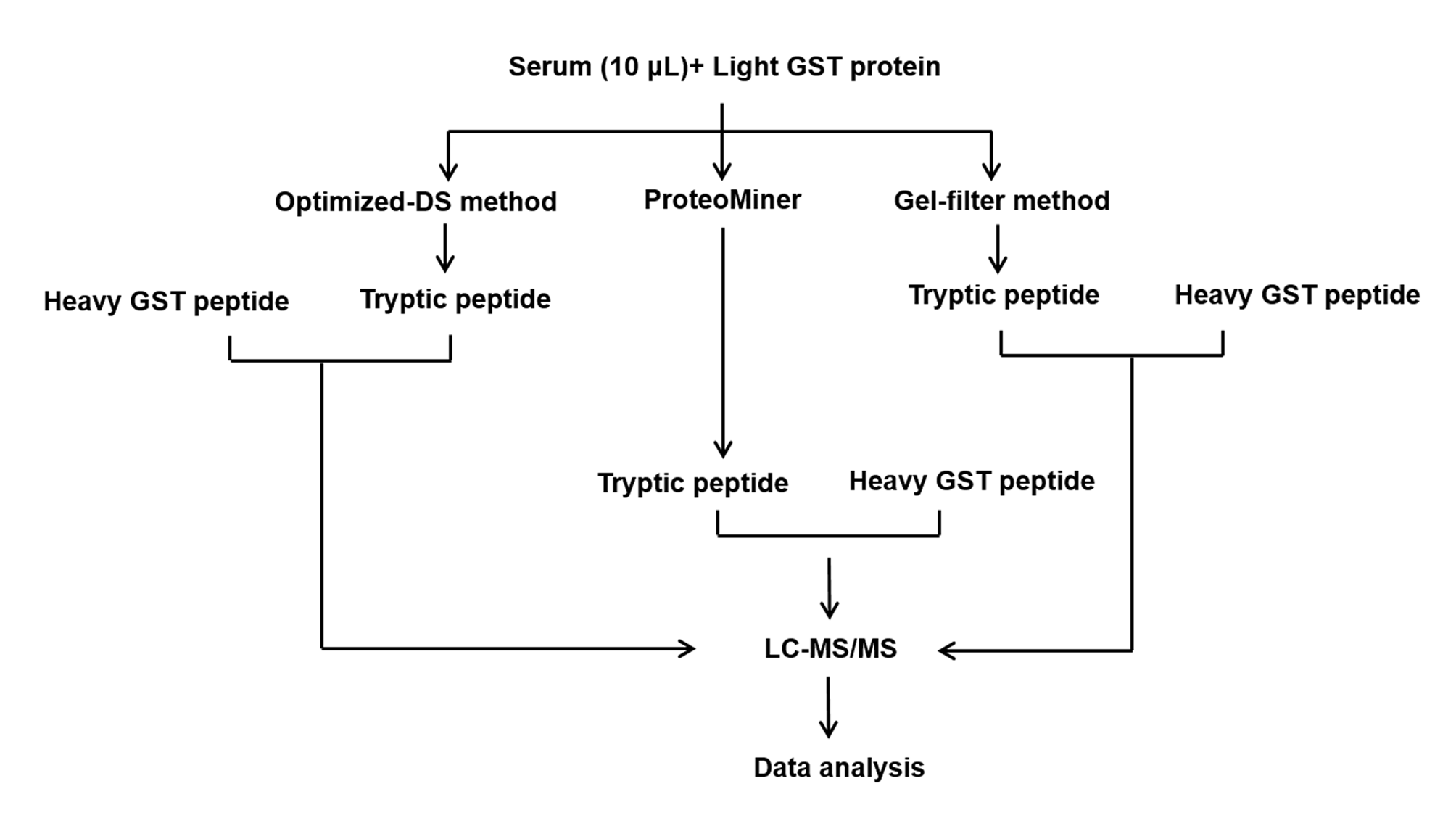

Supplement: S5 Fig — The comparison for recovery rate of LMW proteins was performed through three different approaches, including optimized-DS, ProteoMiner and Gel-filter methods. (TIF) [file pone.0115862.s006.tif]

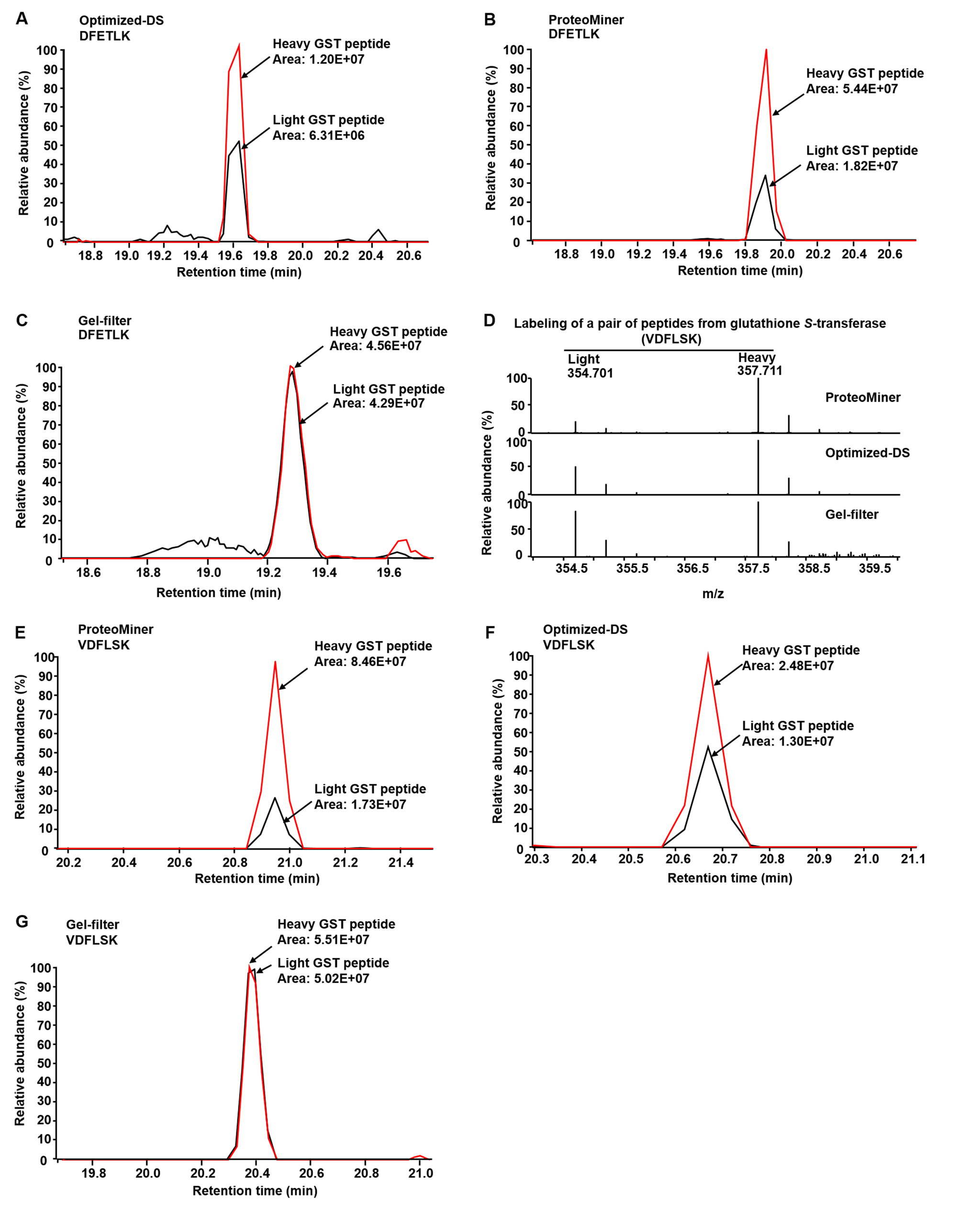

Supplement: S6 Fig — Peptide quantification was performed by SILAC-AQUA methodology. Same amount of peptides digested from light labeled GST was spiked in the samples processed with optimized-DS, ProteoMiner and gel-filter methods, and then analyzed by LC-MS/MS. A and C. Optimized-DS method; E and G. ProteoMiner; I and K. Gel-filter method. Representative spectra with SILAC pairs of the peptide DFETLK and VDFLSK were selected for quantification as indicated. (TIF) [file pone.0115862.s007.tif]
